# Genome Sequence of Bacillus velezensis P1, a Strain Isolated from a Biofilm Captured on a Pig Farm Building

**DOI:** 10.1128/MRA.01219-21

**Published:** 2022-01-27

**Authors:** Virgile Guéneau, Jean-Christophe Piard, Bastien Frayssinet, Valentin Loux, Hélène Chiapello, Julia Plateau-Gonthier, Mathieu Castex, Romain Briandet

**Affiliations:** a Université Paris-Saclay, INRAE, AgroParisTech, Micalis Institute, Jouy-en-Josas, France; b Lallemand SAS, Blagnac, France; c Université Paris-Saclay, INRAE, MaIAGE, Jouy-en-Josas, France; d Université Paris-Saclay, INRAE, BioinfOmics, MIGALE Bioinformatics Facility, Jouy-en-Josas, France; University of Rochester School of Medicine and Dentistry

## Abstract

The genome of the Bacillus velezensis P1 strain isolated from a biofilm on the wall of a pig farm was sequenced. The strain harbors many surface colonization genes involved in surfactant, matrix, and antibacterial synthesis.

## ANNOUNCEMENT

Surface communities on the wall inside a pig farm building were sampled by using coupons installed on the surface for 31 days. Biofilms were recovered by scratching using a pipette cone and 30 mL of saline solution, and the bacterial suspension was treated for 10 min at 80°C to select heat-resistant spores. Plating the treated suspension on Trypticase soy (TS) agar at 30°C for 24 h allowed isolation of the P1 strain forming highly structured colonies on agar.

From a −80°C glycerol stock, the P1 strain was cultured in TS under agitation at 37°C overnight (∼16 h). Genomic DNA was extracted and purified using the Monarch genomic DNA purification kit (New England Biolabs, Ipswich, MA, USA). DNA sequencing (DNA-seq) was performed at the GeT-PlaGe core facility (INRAE, Toulouse, France; www.genotoul.fr/en/). DNA-seq libraries were prepared according to Illumina’s protocols using the TruSeq Nano DNA high-throughout (HT) library kit (Illumina, San Diego, CA, USA). Briefly, DNA was fragmented by sonication, size selection was performed using sample purification beads (SPBs) (kit beads), and adaptors were ligated to be sequenced. Library quality was assessed using the Agilent DNF-474 high-sensitivity (HS) next-generation sequencing (NGS) fragment kit (Agilent Technologies, Santa Clara, CA, USA), and libraries were quantified by quantitative PCR (qPCR) using the Kapa library quantification kit (Roche, Basel, Switzerland). DNA-seq experiments were performed on an Illumina NovaSeq 6000 system, using a paired-end read length of 2 × 150 bp with the Illumina NovaSeq 6000 reagent kits (Illumina). Verification of the libraries was done using NanoDrop quantification (NanoDrop 8000; Thermo Scientific, Waltham, MA, USA). The reads (5,299,802 sequences, sequence length of 150 bp) were analyzed using tools available in Galaxy with default parameters (https://galaxy.migale.inrae.fr/) (1). *De novo* assembly was performed using Unicycler (Galaxy version 0.4.8.0) with quality control done with Quast (Galaxy version 5.0.2 + Galaxy 3) (2, 3), and genome annotation was performed using the Prokaryotic Genome Annotation Pipeline (PGAP, annotation software revision 5.2) (4). The percent genome coverage is 201, and the size of the assembly is 3,901,648 bp, made of 26 contigs with a GC content of 47% and an *N*_50_ value of 564,796 nucleotides. A total of 3,766 predicted protein-coding genes were detected with 78 tRNAs, 3 rRNAs, and 5 noncoding RNAs (ncRNAs). The bacterial species identified with more than 99.9% identity is Bacillus velezensis as determined by a BLAST search of the genome of 100 kb, using the nucleotide database collection of *Bacillus* (taxid: 1386) optimized for highly similar sequences. The Staramr tool (Galaxy version 0.7.2 + Galaxy 0) revealed that *B. velezensis* P1 does not harbor antibiotic resistance genes (https://github.com/phac-nml/staramr) and that no plasmids were predicted.

Different genes involved in biofilm formation were detected in the genome (i.e., *eps*, *sps*, *cap*, *tapA*→*tasA*, *bslA*) (5–7). Using the antiSMASH genome analysis tool, 5 antibacterial genes were detected, encoding with a coverage of more than 98% bacillibactin, macrolactin, bacillaene, difficidin, and bacilysin (8, 9).

The biofilm formation and antimicrobial secretion properties of *B. velezensis* P1 are assumed to confer significant fitness to live and persist in the livestock building surface biotope.

### Data availability.

This whole-genome shotgun project has been deposited at DDBJ/ENA/GenBank under the accession number JAHLGR000000000 and in the NCBI Sequence Read Archive (SRA) under BioProject accession number PRJNA736682. The draft genome assembly and annotation can be found under BioProject number PRJNA736682 and BioSample number SAMN19656044, respectively.

## References

[1] Afgan E, Baker D, Batut B, van den Beek M, Bouvier D, Cech M, Chilton J, Clements D, Coraor N, Grüning BA, Guerler A, Hillman-Jackson J, Hiltemann S, Jalili V, Rasche H, Soranzo N, Goecks J, Taylor J, Nekrutenko A, Blankenberg D. 2018. The Galaxy platform for accessible, reproducible and collaborative biomedical analyses: 2018 update. Nucleic Acids Res 46:W537–W544. doi:10.1093/nar/gky379. 29790989 PMC6030816

[2] Wick RR, Judd LM, Gorrie CL, Holt KE. 2017. Unicycler: resolving bacterial genome assemblies from short and long sequencing reads. PLoS Comput Biol 13:e1005595. doi:10.1371/journal.pcbi.1005595. 28594827 PMC5481147

[3] Mikheenko A, Prjibelski A, Saveliev V, Antipov D, Gurevich A. 2018. Versatile genome assembly evaluation with QUAST-LG. Bioinformatics 34:i142–i150. doi:10.1093/bioinformatics/bty266. 29949969 PMC6022658

[4] Li W, O’Neill KR, Haft DH, DiCuccio M, Chetvernin V, Badretdin A, Coulouris G, Chitsaz F, Derbyshire MK, Durkin AS, Gonzales NR, Gwadz M, Lanczycki CJ, Song JS, Thanki N, Wang J, Yamashita RA, Yang M, Zheng C, Marchler-Bauer A, Thibaud-Nissen F. 2021. RefSeq: expanding the Prokaryotic Genome Annotation Pipeline reach with protein family model curation. Nucleic Acids Res 49:D1020–D1028. doi:10.1093/nar/gkaa1105. 33270901 PMC7779008

[5] Diehl A, Roske Y, Ball L, Chowdhury A, Hiller M, Molière N, Kramer R, Stöppler D, Worth CL, Schlegel B, Leidert M, Cremer N, Erdmann N, Lopez D, Stephanowitz H, Krause E, van Rossum B-J, Schmieder P, Heinemann U, Turgay K, Akbey Ü, Oschkinat H. 2018. Structural changes of TasA in biofilm formation of Bacillus subtilis. Proc Natl Acad Sci USA 115:3237–3242. doi:10.1073/pnas.1718102115. 29531041 PMC5879678

[6] Eichenberger P, Fujita M, Jensen ST, Conlon EM, Rudner DZ, Wang ST, Ferguson C, Haga K, Sato T, Liu JS, Losick R. 2004. The program of gene transcription for a single differentiating cell type during sporulation in Bacillus subtilis. PLoS Biol 2:e328. doi:10.1371/journal.pbio.0020328. 15383836 PMC517825

[7] Earl C, Arnaouteli S, Bamford NC, Porter M, Sukhodub T, MacPhee CE, Stanley-Wall NR. 2020. The majority of the matrix protein TapA is dispensable for Bacillus subtilis colony biofilm architecture. Mol Microbiol 114:920–933. doi:10.1111/mmi.14559. 32491277

[8] Blin K, Shaw S, Steinke K, Villebro R, Ziemert N, Lee SY, Medema MH, Weber T. 2019. antiSMASH 5.0: updates to the secondary metabolite genome mining pipeline. Nucleic Acids Res 47:W81–W87. doi:10.1093/nar/gkz310. 31032519 PMC6602434

[9] Chen XH, Koumoutsi A, Scholz R, Eisenreich A, Schneider K, Heinemeyer I, Morgenstern B, Voss B, Hess WR, Reva O, Junge H, Voigt B, Jungblut PR, Vater J, Süssmuth R, Liesegang H, Strittmatter A, Gottschalk G, Borriss R. 2007. Comparative analysis of the complete genome sequence of the plant growth-promoting bacterium Bacillus amyloliquefaciens FZB42. Nat Biotechnol 25:1007–1014. doi:10.1038/nbt1325. 17704766

